# Zinc finger nuclease‐mediated precision genome editing of an endogenous gene in hexaploid bread wheat (*Triticum aestivum*) using a DNA repair template

**DOI:** 10.1111/pbi.12941

**Published:** 2018-05-28

**Authors:** Yidong Ran, Nicola Patron, Pippa Kay, Debbie Wong, Margaret Buchanan, Ying‐Ying Cao, Tim Sawbridge, John P. Davies, John Mason, Steven R. Webb, German Spangenberg, William M. Ainley, Terence A. Walsh, Matthew J. Hayden

**Affiliations:** ^1^ Genovo Biotechnology Co. Ltd Tianjin China; ^2^ Earlham Institute Norwich Science Park Norfolk UK; ^3^ Department of Economic Development, Jobs, Transport and Resources Centre for AgriBioscience Agriculture Victoria Research AgriBio Bundoora Vic. Australia; ^4^ School of Applied Biology La Trobe University Bundoora Vic. Australia; ^5^ Dow AgroSciences LLC Indianapolis IN USA

**Keywords:** zinc finger nuclease, genome editing, nonhomologous end‐joining, herbicide tolerance, acetohydroxyacid synthase, wheat

## Abstract

Sequence‐specific nucleases have been used to engineer targeted genome modifications in various plants. While targeted gene knockouts resulting in loss of function have been reported with relatively high rates of success, targeted gene editing using an exogenously supplied DNA repair template and site‐specific transgene integration has been more challenging. Here, we report the first application of zinc finger nuclease (ZFN)‐mediated, nonhomologous end‐joining (NHEJ)‐directed editing of a native gene in allohexaploid bread wheat to introduce, via a supplied DNA repair template, a specific single amino acid change into the coding sequence of *acetohydroxyacid synthase* (*AHAS*) to confer resistance to imidazolinone herbicides. We recovered edited wheat plants having the targeted amino acid modification in one or more AHAS homoalleles via direct selection for resistance to imazamox, an AHAS‐inhibiting imidazolinone herbicide. Using a cotransformation strategy based on chemical selection for an exogenous marker, we achieved a 1.2% recovery rate of edited plants having the desired amino acid change and a 2.9% recovery of plants with targeted mutations at the AHAS locus resulting in a loss‐of‐function gene knockout. The latter results demonstrate a broadly applicable approach to introduce targeted modifications into native genes for nonselectable traits. All ZFN‐mediated changes were faithfully transmitted to the next generation.

## Introduction

Several technologies for genome engineering have been demonstrated in plant species including agriculturally important crops such as maize, rice, wheat, tomato, cotton, soybean and barley (D'Halluin *et al*., 2013; Lawrenson *et al*., 2015; Li *et al*., 2015, 2016; Shukla *et al*., 2009; Svitashev *et al*., 2015; Wang *et al*., 2014). These technologies use engineered nucleases such as meganucleases, zinc finger nucleases (ZFNs), transcription activator‐like effector nucleases (TALENs) or RNA‐guided Cas9 nucleases from bacterial clustered regularly interspaced short palindromic repeats (CRISPR) systems (reviewed in Belhaj *et al*., 2015; Luo *et al*., 2016; Schaeffer and Nakata, 2016; Zhu *et al*., 2017) to induce double‐stranded DNA breaks (DSBs) at a precise position in the genome. Creation of a DSB stimulates the native cellular DNA repair mechanisms, which can be used to engineer the target locus to result in targeted mutagenesis, precision editing to a desired sequence or targeted integration of new DNA (Voytas, 2013). Targeted integration offers several advantages over traditional transgenic approaches that rely on random integration of the transgene into the genome: it has the potential to enable the evaluation of different transgene constructs at the same genetic locus, reducing the position effect known to affect expression levels (Srivastava *et al*., 2004), and it reduces commercial breeding challenges for generating varieties with transgene stacks by enabling multiple transgenes to be positioned at the same genomic position for transmission as a single Mendelian locus (Ainley *et al*., 2013; Kumar *et al*., 2016). Targeted mutagenesis provides an alternative to traditional mutagenesis methods such as ethyl methane sulphonate and irradiation for forward genetic screens and for reverse genetic methods such as TILLING (McCallum *et al*., 2000) that rely on target DNA screening of heavily mutagenized populations (see, e.g. Braatz *et al*., 2017; Jacobs *et al*., 2017; Ma *et al*., 2015).

The two main cellular mechanisms for DSB repair in eukaryotic cells are nonhomologous end‐joining (NHEJ) and homology‐directed repair (HDR). NHEJ is generally active throughout the cell cycle and the DNA ligase IV‐mediated rejoining of DNA can be error‐prone, resulting in small insertions or deletions at the site of the break, or in some cases, nucleotide changes (Hiom, 2010). NHEJ can therefore be used to create targeted gene knockouts (Endo *et al*., 2015; Gao *et al*., 2010; Lawrenson *et al*., 2015; Li *et al*., 2012). However, it has also been used for targeted gene insertions (Maresca *et al*., 2013), replacements (Li *et al*., 2016) and genomic deletions (Gao *et al*., 2015; Qi *et al*., 2013; Yoshimi *et al*., 2016; Zhou *et al*., 2014). In contrast, HDR is typically a high‐fidelity process that uses a DNA template with homology to a genomic target sequence and is mainly active in the late S and G2 phase of the cell cycle (Hiom, 2010). In plants, the capacity for HDR is known to substantially decrease as plants mature because fewer cells are rapidly dividing (Boyko *et al*., 2006). HDR can be exploited for targeted gene editing, insertion and replacement by simultaneous provision of a repair DNA template with regions that are homologous to the sequences flanking the induced DSB (Budhagatapalli *et al*., 2015; D'Halluin *et al*., 2013; Steinert *et al*., 2016).

To date, the application of engineered nucleases for targeted genome modification in plants has been mostly reported for targeted gene knockout, which exploits the error‐prone NHEJ repair pathway to generate loss‐of‐function alleles. Relatively high rates of success are reported, with the rate of recovery of plants with loss‐of‐function alleles ranging from 2% to 75%, with a median of 25% (reviewed in Zhu *et al*., 2017). The creation of targeted gene knockouts requires only the delivery of an engineered nuclease to the plant cell. In contrast, targeted gene‐editing and site‐specific transgene integration, which requires codelivery of an exogenous DNA repair template with the engineered nuclease, is reported less frequently and with lower rates of success. Typically, the rate of recovery of plants with targeted modifications derived from NHEJ‐ or HR‐directed DNA repair using a donor template ranges from 0.3% to 15%, with a median of 1.5% (D'Halluin *et al*., 2013; Li *et al*., 2016; Shukla *et al*., 2009; Svitashev *et al*., 2015; Wang *et al*., 2014; Zhu *et al*., 2017). This reduced recovery rate may reflect inherent challenges associated with recruiting the native cellular pathway needed to deliver a specific type of targeted modification, the ability of the cellular repair machinery to access an exogenously supplied template during the DSB repair and the design of the repair template itself. While several studies (e.g. Kan *et al*., 2017; Orlando *et al*., 2010) have investigated the molecular basis for targeted modifications derived from NHEJ‐ and HR‐directed DNA repair using an exogenous DNA repair template in plants, it is currently unclear how the importance of such factors might change between plant species, and even within a plant species when different types of explant are used for plant transformation. Consequently, the generation of targeted gene edits using an exogenously supplied DNA repair template and site‐specific transgene integration remains a challenge in plants.

Zinc finger nuclease have been used in plants for targeted chromosomal deletions (Lee *et al*., 2010; Söllü *et al*., 2010), transgene removal (Petolino *et al*., 2010; Weinthal *et al*., 2013) and targeted DNA integration (Ainley *et al*., 2013; Shukla *et al*., 2009). ZFNs are essentially engineered restriction enzymes designed to precisely bind and cleave user‐specified DNA sequences. They consist of a nonspecific cleavage domain from the type IIS restriction endonuclease *Fok*I fused to an array of 4–6 zinc finger protein domains that each recognize about three bp of DNA (Urnov *et al*., 2010). ZFNs have high specificity as dimerization of the *Fok*I nuclease domain is required to create a DSB, which requires two ZFN monomers to orient themselves with appropriate spacing at the target site (for reviews, see Urnov *et al*., 2010; Petolino, 2015; Tzfira *et al*., 2012). *FokI* variants requiring heterodimerization between the ZFN monomers comprising the ZFN pair have also been developed to further enhance the sequence specificity of ZFNs and to reduce off‐site cleavage due to homodimerization of individual ZFN monomers (Doyon *et al*., 2011).

Acetohydroxyacid synthase ‐inhibiting herbicides such as imidazolinones and sulfonylureas are important for weed control in farming systems. These herbicides target acetohydroxyacid synthase (AHAS, EC 2.2.1.6), which catalyses a key step in the synthesis of branched chain amino acids (Tan *et al*., 2006). Inhibition of AHAS leads to rapid cessation of growth and subsequent plant death (Shaner *et al*., 1984). Five single amino acid changes that do not affect the catalytic activity of AHAS but reduce sensitivity to herbicides have been described (Yu and Powles, 2014), and resistant varieties for several crop species have been successfully produced through mutagenesis approaches (Andersson *et al*., 2003; Pozniak *et al*., 2004; Swanson *et al*., 1989; Walter *et al*., 2014). Allohexaploid wheat (*Triticum aestivum* L., 2n = 42, AABBDD) has a single copy of the *AHAS* gene in each of chromosomes 6A, 6B and 6D and the occurrence of a single amino acid change from serine to arginine in the AHAS protein at position 630 (equivalent to position 653 in the orthologous AHAS protein in *Arabidopsis*) confers strong resistance to imidazolinones (Pozniak *et al*., 2004).

In this study, we targeted the three homoeologous copies of *AHAS* in bread wheat to demonstrate that ZFNs can facilitate simultaneous multiple gene knockouts and desired native gene sequence changes via NHEJ‐directed DNA repair in a polyploid crop. To achieve this, we cotransformed wheat plant cells with an exogenously supplied DNA repair template designed to utilize the NHEJ repair pathway and a ZFN targeting the three *AHAS* homoeologous. Using two transformation strategies, we demonstrate the ability of ZFNs to efficiently create precise genome modifications at both selectable and nonselectable trait loci. Our analysis of *T*_1_ plants confirmed that gene knockouts and designed gene edits facilitated by ZFNs are heritable and show the expected Mendelian segregation.

## Results

### Characterization of ZFNs targeting endogenous AHAS genes in wheat

**Figure 1:**
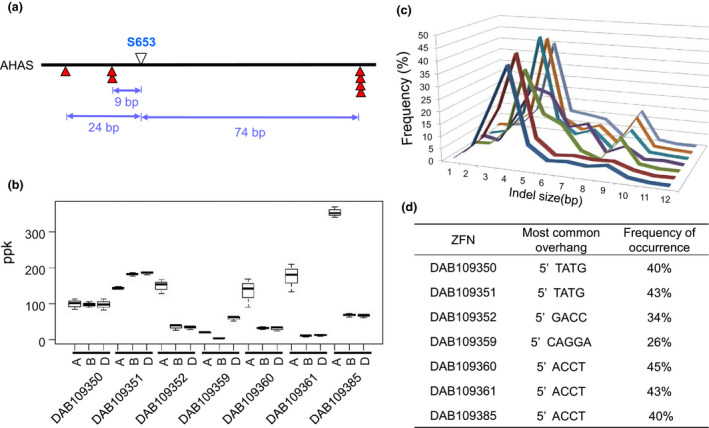
Position and activity of ZFNs. (a) Distance of ZFN cleavage sites in base pairs from the S653 residue in the AHAS protein; (b) observed ZFN efficacy on each AHAS homoeolog, calculated as the number of sequence reads with indels divided by the total number of reads for a given gene target and expressed as parts per thousand (ppk) activity after normalizing for protoplast transfection efficiency. Results are from three biological replicates; (c) Frequency distribution of indel sizes used to determine the size of the predominant overhang generated by each ZFN. Order of ZFNs (from front to back) is same as that shown in the table in this figure caption; (d) predominant deduced overhang generated by each ZFN.

PCR amplification, cloning and Sanger sequencing of the *AHAS* gene homoeologous (*AHAS‐6A, AHAS‐6B* and *AHAS‐6D*) in the bread wheat genotype Bobwhite MBP26RH revealed about 97% and 98% identity at the nucleotide and protein levels, respectively. *In silico* ZFN design for the *AHAS* gene region encoding the S630 residue (hereafter referred to as S653 to maintain consistency with *Arabidopsis* nomenclature) in the protein sequence generated seven ZFNs predicted to cleave all three homoeologous gene copies. Three of the ZFN cut sites were located upstream of the S653 codon, while the remainder were positioned downstream. The ZFN cut site designed to be closest to the target was predicted to create a DSB nine‐bp upstream of the S653 residue (Figure 1a).

The efficacy of each ZFN was tested against their endogenous targets using transient assays based on PEG‐mediated DNA delivery to wheat mesophyll protoplasts. ZFN efficacy was assessed by measuring whether they created small insertion and deletion (indel) mutations by nonhomologous end joining (NHEJ), which has previously been shown to provide a reliable indicator for ZFN activity at endogenous loci (Shukla *et al*., 2009; Townsend *et al*., 2009). Analysis of the proportion of sequence reads showing indel mutations revealed that all ZFNs tested had activity in protoplasts and were able to create targeted DSBs in each of the homoeologous copies of the *AHAS* gene. Some ZFNs showed differential cleavage activity across *AHAS* homoeologous, while others had similar activity (Figure 1b). Examination of the frequency and pattern of indel mutations for each ZFN allowed the size of the overhangs generated by *Fok*I cleavage at the ZFN binding site to be accurately determined. The most common deletion for the ZFNs was four bp, resulting from trimming of the single‐stranded ends of the overhangs (Figure 1c).

### Efficiencies for NHEJ‐directed gene editing in wheat

To examine the efficiency for ZFN‐mediated NHEJ‐directed genome editing in wheat, two strategies for allele editing via a supplied DNA repair template (hereafter called a donor) were tested using transient assays based on particle bombardment DNA delivery to scutella of immature zygotic embryos: allele insertion and allele replacement. Our choice of tissue for transformation experiments was based on the current inability to reliably regenerate plants from wheat mesophyll protoplasts, making it necessary to use the scutella system to recover stable transformation events.

**Figure 2:**
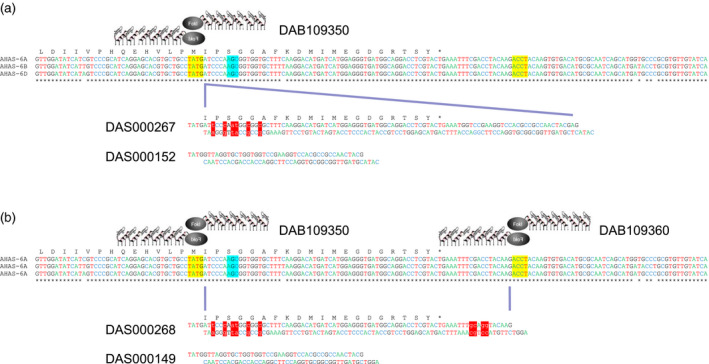
Targeted editing of wheat AHAS genes using: (a) allele insertion and (b) allele replacement. Donor mutations relative to native AHAS sequence are depicted by red highlight and lowercase text. ZFN generated overhangs at DSBs are shown in yellow. The position of the codon for the S653 amino acid in the native AHAS gene is indicated by cyan highlight.

The strategy for allele insertion – the introduction of new DNA in‐frame with the existing gene – used a single ZFN to induce a DSB located nine‐bp upstream of the S653 codon in the *AHAS* homoeologous. The strategy for allele replacement – the seamless replacement of endogenous *AHAS* sequence with new sequence – used a pair of ZFNs to induce DSBs either side of the S653 codon. These ZFNs induced DSBs positioned 9‐ and 74‐bp upstream and downstream of the S653 codon, respectively. Both strategies employed the codelivery of a 41‐bp double‐stranded linear donor DNA fragment to be inserted into ZFN‐induced DSBs in the three homoeologous copies of the endogenous *AHAS* gene. As *Fok*I digestion results in the generation of 5′ overhangs, each donor repair template was produced with specific 5′ overhangs to facilitate error‐free ligation of the donor into the DSB created by the ZFN(s). Donor DAS000152 was designed to provide ligation overhangs compatible with those generated by cleavage of the endogenous *AHAS* genes by ZFN DAB109350 and to result in insertion of the 41‐bp donor fragment at the site of the endogenous DSB (Figure 2a). Donor DAS000149 had ligation overhangs compatible with those generated by dual cleavage of the endogenous *AHAS* genes by ZFNs DAB109350 and DAB109360 and was designed to replace the intervening endogenous *AHAS* sequence contained between the two induced DSBs (Figure 2b).

Deep sequencing of amplicons generated by PCR amplification with primers flanking the entire target site in the endogenous *AHAS* genes was used to assess gene‐editing outcomes in scutella cotransformed with the donor DNA and ZFN encoding plasmid(s), or with donor molecule only.

As particle bombardment‐mediated methods deliver DNA to only a small number of scutella cells, the vast majority of cells are expected to contain unmodified *AHAS* gene sequences. We therefore assessed editing frequency by calculating the percentage of sequence reads showing evidence for integration of the donor DNA at the targeted DSB as a proportion of the total number of reads showing evidence of ZFN‐mediated mutations. We also determined the proportion of reads showing evidence for perfect and imperfect donor integration, where perfect integration denotes seamless donor integration at the endogenous DSB and imperfect integration indicates the additional presence of indels caused by NHEJ‐associated exonuclease activity at the repair junctions.

About 0.02% of sequence reads generated from scutella cotransformed with ZFN DAB109350 and donor DAS000152 showed evidence for ZFN‐induced indel mutations or donor integration. About 36% of these reads showed evidence for donor integration, while the remainder had indel mutations. The majority (86%) of reads with donor integration had perfect gene editing. The frequency of observed gene‐editing outcomes was similar across the A, B and D genomes. No reads with donor integration were observed for scutella transformed only with donor DAB000152 (Table 1). Of the sequence reads generated for scutella cotransformed with ZFNs DAB109350 and DAB109360 and donor DAS000149, about 0.006% showed evidence for ZFN‐induced indels and donor integration. About 6% of these reads showed evidence for donor integration, while the remainder had indel mutations. Twenty‐two (219/1010) per cent of the reads containing indels showed evidence for cleavage at both ZFN sites and loss of the intervening sequence between the DSBs; the remainder (78%; 791/1010) had an indel corresponding to the creation of a DSB by only one ZFN. The vast majority (99%) of reads with donor integration had perfect gene editing. Again, the frequency of observed gene‐editing outcomes was similar across the three wheat subgenomes. No reads with donor integration were observed for scutella transformed only with donor DAS000149 (Table 1).

**Table 1:** Per cent of sequence reads showing evidence for perfect and imperfect gene integration and replacement

Vectors	Homoeolog	Total reads	No. of reads containing indel at single ZFN cleavage site	No. of reads containing indel at both ZFN cleavage sites	No. of reads containing donor	% donor‐containing reads	No. of donor‐containing reads without indels	% donor‐containing reads without indels
ZFN DAB109350 + donor DAS000152	AHAS‐6A	4 277 922	663	na	562	46	452	80
ZFN DAB109350 + donor DAS000152	AHAS‐6B	8 969 947	1058	na	424	29	368	87
ZFN DAB109350 + donor DAS000152	AHAS‐6D	7 424 334	1130	na	555	33	498	90
	Average	6 890 734	950	na	514	36	439	86
Donor DAS000152	AHAS‐6A	2 353 804	1	na	0	0	0	0
Donor DAS000152	AHAS‐6B	4 724 973	2	na	0	0	0	0
Donor DAS000152	AHAS‐6D	4 197 708	7	na	0	0	0	0
	Average	3 758 828	3	na	0	0	0	0
ZFNs DAB109350 and DAB109360 + donor DAS000149	AHAS‐6A	2 863 426	521	153	67	9	67	100
ZFNs DAB109350 and DAB109360 + donor DAS000149	AHAS‐6B	5 647 765	733	149	42	5	42	100
ZFNs DAB109350 and DAB109360 + donor DAS000149	AHAS‐6D	6 318 945	1118	356	47	3	46	98
	Average	4 943 379	791	219	52	6	52	99
Donor DAS000149	AHAS‐6A	1 866 240	0	14	0	0	0	0
Donor DAS000149	AHAS‐6B	3 909 015	13	3	0	0	0	0
Donor DAS000149	AHAS‐6D	3 385 921	8	2	0	0	0	0
	Average	3 053 725	7	6	0	0	0	0

### Generation and characterization of wheat plants with AHAS(S653N) via direct selection

To demonstrate ZFN‐mediated NHEJ‐directed editing of an endogenous gene to create a selectable trait, the *AHAS* genes in wheat were targeted for the introduction of the S653N mutation known to confer resistance to imidazolinone class herbicides. Scutella of immature zygotic wheat embryos were cotransformed with ZFN encoding plasmid and a linear double‐stranded DNA donor using particle bombardment. Two sets of cotransformation were performed. The first delivered ZFN DAB109350 and donor DAS000267, while the second delivered ZFNs DAB109350 and DAB109360 and donor DAS000268 (Figure 2). Four selection schedules were used to recover stably transformed *T*_0_ wheat plants having resistance to imazamox, an imidazolinone class herbicide (Table 2).

**Table 2:** Selection strategies used to recover AHAS‐edited wheat plants with imazamox resistance

Plant Regeneration Stage	Strategy 1	Strategy 2	Strategy 3	Strategy 4
Callus Induction	150	250	150	250
Plant Regeneration	150	0	250	250
Rooting	200	200	200	200

All values are in nM imazamox in the media.

The double‐stranded 95‐bp donor DAS000267 (Figure 2) was designed to integrate via the NHEJ pathway at a DSB created in an *AHAS* homoeolog by ZFN DAB109350 and comprised two parts. The 5′ end contained sequence homologous to the endogenous *AHAS* gene encoded in the D genome, starting from the target ZFN cleavage site and ending at the *AHAS* stop codon but containing six intentional base changes: two mutations encoded the S653N mutation (AGC‐>AAT) and four synonymous base changes to prevent ZFN recognition and cleavage of the integrated donor. The 3′ end of the donor contained a unique 28‐bp sequence that could be used for diagnostic PCR to detect the integrated donor in regenerated wheat plants. The donor molecule had protruding 5′ ends to provide ligation overhangs compatible with those produced by ZFN DAB109350.

The double‐stranded 79‐bp donor DAS000268 (Figure 2) was designed to replace the intervening sequence between the cleavage sites in the endogenous *AHAS* genes for ZFNs DAB109350 and DAB109360. The donor contained sequence homologous to the endogenous *AHAS* gene encoded in the D genome, starting from the cleavage site for ZFN DAB109350 and ending at the cleavage site for ZFN DAB109360 but contained ten intentional base changes. Six base changes were located at the 5′ end of the donor: two encoded the S653N mutation (AGC‐>AAT) and four encoded synonymous mutations. Four base changes were located at the 3′ end of the donor and positioned in noncoding sequence. The additional base changes were again used to prevent ZFN recognition and cleavage of integrated donor. The donor molecule had protruding 5′ ends to provide ligation overhangs compatible with those generated by the pair of ZFNs.

**Figure 3:**
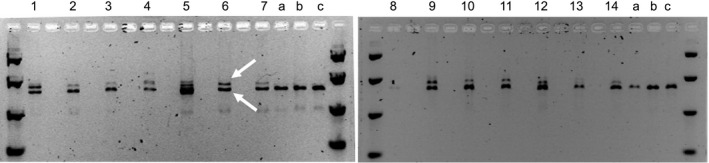
Outcome of PCR analysis performed on regenerated *T*_0_ wheat plants: (1–12) allele insertion events derived from transformation with ZFN DAB109350 and donor DAS000267; (13–14) allele replacement events derived from transformation with ZFNs DAB109350 and DAB109360 and donor DAS000268; (a‐c) wild‐type control plants. Lower and upper arrows indicate allele insertion and wild‐type allele, respectively.

Cobombardment of about 4000 scutella with ZFN DAB109350 and donor DAS000267 for each imazamox selection strategy resulted in the regeneration of 12 wheat seedlings, with 2–5 plants recovered from each strategy (Table 3). PCR amplification across the target sites in the endogenous *AHAS* genes confirmed allele insertion via stable donor integration at the target site in at least one of the endogenous *AHAS* genes in each of the recovered wheat plants (Figure 3).

**Table 3:** Recovery of transformed *T*_0_ wheat plants with ZFN‐mediated allele insertion and replacement

	Allele insertion				Allele replacement			
	Strategy 1	Strategy 2	Strategy 3	Strategy 4	Strategy 1	Strategy 2	Strategy 3	Strategy 4
No. bombarded scutella	4080	3740	4000	3800	2020	1790	1840	1660
No. IMI tolerant wheat plants	5	3	2	2	0	2	0	0

For deliveries with ZFNs DAB109350 and DAB109360 and donor DAS000268, about 2000 scutella were cobombarded for each imazamox selection strategy. Two wheat seedlings were regenerated from the second selection strategy (Table 3), both of which were confirmed by PCR analysis to have stable donor integration in at least one copy of the endogenous *AHAS* genes. However, the PCR results for both wheat seedlings suggested the intervening endogenous sequence between the two ZFN binding sites had not been excised, as a larger‐than‐expected PCR fragment was observed (Figure 3). Excision of intervening endogenous *AHAS* sequence was expected to result in the amplification of only wild‐type fragment sizes.

To characterize the subgenomic location and gene‐editing outcomes in the 14 regenerated *T*_0_ wheat seedlings, the PCR products derived from amplification across the target sites in the *AHAS* gene homoeologous were cloned and Sanger sequenced. Up to 120 independent plasmid clones from each *T*_0_ plant were sequenced to ensure each *AHAS* homoeoallele was characterized. A consensus sequence for each allele was generated and used to determine the subgenomic origin and sequence for each edited allele.

**Figure 4:**
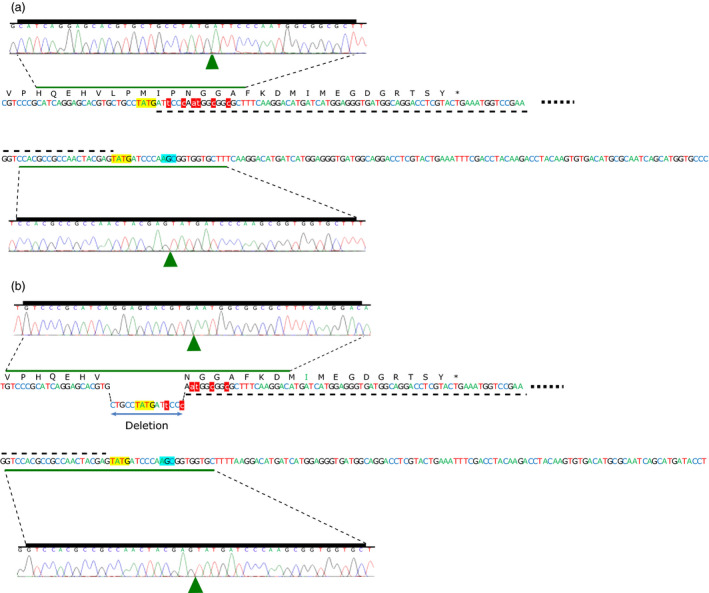
NHEJ‐mediated allele insertion of two AHAS homoeologous in *T*_0_ Event 1. (a) Perfect donor integration at both junctions of the DSB in the A genome; (b) Imperfect donor integration due to the presence of a 15‐bp in‐frame deletion at the 5′ junction of the DSB break in the B genome. Green triangles denote junctions of DSB in the Sanger traces. Dashed lines indicate integrated donor sequence, while red highlighted and lowercase text indicates donor mutations relative to native AHAS sequence. ZFN generated overhangs are shown in yellow highlight. The position of the codon for the S653 amino acid in the native AHAS gene is indicated by cyan highlight.

Targeted allele insertion at the endogenous *AHAS* loci was detected for 11 of the 12 regenerated *T*_0_ wheat plants that were transformed using ZFN DAB109350 and donor DAS000267 (Table 4). The remaining plant (Event 8) contained only wild‐type alleles and was considered an escape from selection. The 11 insert‐positive plants all contained at least one allele spliced gene that was predicted to produce a functional AHAS protein containing the S653N mutation, and explained the observed resistance of the wheat plant to imazamox. The 11 plants showed a range of gene‐editing outcomes including (i) perfect monoallelic allele insertion in the A, B or D genome, for example Events 6, 10 and 11; (ii) simultaneous allele insertion in multiple subgenomes, for example Events 1, 4 and 9; (iii) perfect biallelic allele insertion, for example Event 9; and (iv) different biallelic editing outcomes at the same locus, for example Events 1, 4 and 12. Examples of *T*_0_ plants with perfect and imperfect allele insertion are shown in Figure 4.

**Table 4:** AHAS‐editing outcomes observed in *T*_0_ wheat plants recovered from transformations with: (1–12) ZFN DAB109350 and donor DAS000267; and (13–14) ZFNs DAB109350 and DAB109360 and donor DAS000268

Event		A genome		B genome		D genome	
		Allele 1	Allele 2	Allele 1	Allele 2	Allele 1	Allele 2
1	Editing outcome	AI	KO	AI	WT	AI	WT
	Functional AHAS(S653N) protein	Yes	.	No	.	No	.
	5′ junction of DSB	.	Indel	Indel	.	Indel	.
	3′ junction of DSB	.	.	.	.	Indel	.
2	Editing outcome	KO	WT	WT	WT	AI	WT
	Functional AHAS(S653N) protein	.	.	.	.	Yes	.
	5′ junction of DSB	Indel	.	.	.	.	.
	3′ junction of DSB	.	.	.	.	Indel	.
3	Editing outcome	AI	WT	WT	WT	WT	WT
	Functional AHAS(S653N) protein	Yes	.	.	.	.	.
	5′ junction of DSB	.	.	.	.	.	.
	3′ junction of DSB	.	.	.	.	.	.
4	Editing outcome	AI	WT	AI	WT	AI	AI
	Functional AHAS(S653N) protein	Yes	.	No^a^	.	Yes	Yes^b^
	5′ junction of DSB	.	.	Indel	.	.	.
	3′ junction of DSB	.	.	Indel	.	.	.
5	Editing outcome	AI	WT	KO	WT	WT	WT
	Functional AHAS(S653N) protein	Yes	.	.	.	.	.
	5′ junction of DSB	.	.	Indel	.	.	.
	3′ junction of DSB	.	.	.	.	.	.
6	Editing outcome	WT	WT	AI	WT	WT	WT
	Functional AHAS(S653N) protein	.	.	Yes	.	.	.
	5′ junction of DSB	.	.	.	.	.	.
	3′ junction of DSB	.	.	.	.	.	.
7	Editing outcome	AI	WT	WT	WT	WT	WT
	Functional AHAS(S653N) protein	Yes	.	.	.	.	.
	5′ junction of DSB	.	.	.	.	.	.
	3′ junction of DSB	.	.	.	.	.	.
8	Editing outcome	WT	WT	WT	WT	WT	WT
	Functional AHAS(S653N) protein	.	.	.	.	.	.
	5′ junction of DSB	.	.	.	.	.	.
	3′ junction of DSB	.	.	.	.	.	.
9	Editing outcome	AI	AI	AI	WT	WT	WT
	Functional AHAS(S653N) protein	Yes	Yes	No	.	.	.
	5′ junction of DSB	.	.	Indel	.	.	.
	3′ junction of DSB	.	.	.	.	.	.
10	Editing outcome	AI	WT	WT	WT	WT	WT
	Functional AHAS(S653N) protein	Yes	.	.	.	.	.
	5′ junction of DSB	.	.	.	.	.	.
	3′ junction of DSB	.	.	.	.	.	.
11	Editing outcome	WT	WT	WT	WT	AI	WT
	Functional AHAS(S653N) protein	.	.	.	.	Yes	.
	5′ junction of DSB	.	.	.	.	.	.
	3′ junction of DSB	.	.	.	.	.	.
12	Editing outcome	WT	WT	WT	WT	AI	KO
	Functional AHAS(S653N) protein	.	.	.	.	Yes	
	5′ junction of DSB	.	.	.	.	.	Indel
	3′ junction of DSB	.	.	.	.	Indel	.
13	Editing outcome	AI	WT	WT	WT	AI	WT
	Functional AHAS(S653N) protein	Yes	.	.	.	Yes	.
	5′ junction of DSB	.	.	.	.	.	.
	3′ junction of DSB	Indel	.	.	.	Indel	.
14	Editing outcome	AI	WT	WT	WT	WT	WT
	Functional AHAS(S653N) protein	Yes	.	.	.	.	.
	5′ junction of DSB	.	.	.	.	.	.
	3′ junction of DSB	Indel	.	.	.	.	.

AI, allele insertion; KO, ablated (knockout) allele; WT, wild‐type allele. Decimal (.) indicates no change relative to wild‐type

a Complex donor integration that could not be fully resolved by the methods used in the analysis.

b Incomplete tandem donor integration.

Similarly, both of the regenerated wheat plants that were transformed using ZFNs DAB109350 and DAB109360 and donor DAS000268 (Table 4) had targeted gene editing. One plant had monoallelic editing in the A and D genomes, while the other plant had monoallelic editing in the A genome. Neither plant had the expected allele replacement but rather showed allele insertion via donor integration.

### Generation and characterization of wheat plants with AHAS(S653N) via indirect selection

The previous experiments demonstrated that direct gene editing to confer a selectable herbicide resistance trait in wheat was feasible across multiple alleles. To demonstrate the feasibility for ZFN‐mediated NHEJ‐directed editing using a supplied DNA repair template at a nonselectable locus, scutella from immature zygotic wheat embryos were cotransformed with ZFN DAB109350, donor DAS000267 and a plasmid encoding a wheat codon optimized version of the *phosphinothricin acetyl transferase* (PAT) gene. This allowed the selection and regeneration of stably transformed wheat plants using the selection agent phosphinothricin.

Cobombardment of 2320 embryos led to the regeneration of 170 stably transformed wheat plants selected using phosphinothricin. PCR performed across the target sites in the endogenous *AHAS* genes confirmed two (1.2%) of the transformed wheat plants had integrated donor at one or more of the *AHAS* loci. Digital droplet PCR performed to detect ZFN‐induced indels identified an additional five (2.9%) *T*_0_ plants with modified *AHAS* alleles. Clones of the two wheat plants with integrated donor were tested for their ability to produce roots when grown on rooting media containing imazamox and were confirmed to be resistant to the AHAS inhibitor.

Characterization of the subgenomic location and nature of gene modifications in the seven wheat plants by sequencing of PCR products derived from amplification across the *AHAS* target sites confirmed that the two wheat plants showing imazamox resistance had at least one functional gene copy encoding the AHAS (S653N) protein (Table 5). Of the remaining plants, four had NHEJ‐derived indels in one of the alleles in the A genome, and one had indels in one allele in the each of the B and D genomes (Table 5).

**Table 5:** AHAS‐editing outcomes observed in *T*_0_ wheat plants recovered from transformations with ZFN DAB109350, donor DAS000267 and a plasmid encoding a wheat codon optimized version of the phosphinothricin acetyl transferase gene (PAT)

Event		A genome		B genome		D genome	
		Allele 1	Allele 2	Allele 1	Allele 2	Allele 1	Allele 2
15	Editing outcome	WT	WT	AI	WT	WT	WT
	Functional AHAS(S653N) protein	.	.	Yes	.	.	.
	5′ junction of DSB	.	.	.	.	.	.
	3′ junction of DSB	.	.	.	.	.	.
16	Editing outcome	AI	AI	WT	WT	WT	WT
	Functional AHAS(S653N) protein	Yes	No	.	.	.	.
	5′ junction of DSB	.	Indel	.	.	.	.
	3′ junction of DSB	Indel	Indel	.	.	.	.
17	Editing outcome	KO	WT	WT	WT	WT	WT
	Functional AHAS(S653N) protein	.	.	.	.	.	.
	5′ junction of DSB	Indel	.	.	.	.	.
	3′ junction of DSB	.	.	.	.	.	.
18	Editing outcome	KO	WT	WT	WT	WT	WT
	Functional AHAS(S653N) protein	.	.	.	.	.	.
	5′ junction of DSB	Indel	.	.	.	.	.
	3′ junction of DSB	.	.	.	.	.	.
19	Editing outcome	KO	WT	WT	WT	WT	WT
	Functional AHAS(S653N) protein	.	.	.	.	.	.
	5′ junction of DSB	Indel	.	.	.	.	.
	3′ junction of DSB	.	.	.	.	.	.
20	Editing outcome	KO	WT	WT	WT	WT	WT
	Functional AHAS(S653N) protein	.	.	.	.	.	.
	5′ junction of DSB	Indel	.	.	.	.	.
	3′ junction of DSB	.	.	.	.	.	.
21	Editing outcome	WT	WT	KO	WT	KO	WT
	Functional AHAS(S653N) protein	.	.	.	.	.	.
	5′ junction of DSB	.	.	Indel	.	Indel	.
	3′ junction of DSB	.	.	.	.	.	.

AS, allele insertion; KO, ablated (knockout) allele; WT, wild‐type allele. Decimal (.) indicates no change relative to wild‐type

### Mendelian inheritance of AHAS gene edits

To investigate whether the ZFN‐induced gene edits and indels could be stably transmitted to the next generation, the *T*_0_ wheat seedlings regenerated from the direct and indirect selection transformation strategies were allowed to self‐pollinate and their *T*_1_ progenies were analysed. Digital droplet PCR assays designed to measure the number of wild‐type, indel and donor‐integrated *AHAS* alleles present in each plant showed the expected Mendelian inheritance in the *T*_1_ generation for each selfed *T*_0_ plant. These results were further confirmed by sequencing the PCR products derived from amplification across the target sites in the *AHAS* gene homoeologous for *T*_1_ plants derived from *T*_0_ plants having more than one *AHAS* gene edit or indel (Table S2). PCR analysis for the presence of integrated ZFN expression vector was not performed.

To confirm stable inheritance of herbicide resistance, *T*_1_ seed from each *T*_0_ plant with gene editing resulting in a functional AHAS(S653N) protein was germinated in tissue culture, genotyped and transferred to rooting media containing imazamox after removing the seedlings roots. After 2 weeks of growth on rooting media, *T*_1_ plantlets having at least one *AHAS*(S653N) allele grew long roots and had normal phenotypes. Plantlets lacking any *AHAS*(S653N) alleles had very short roots and became chlorotic, a phenotype also observed for control wild‐type plants (data not shown).

By testing *T*_1_ seedlings derived from wheat plants generated via the indirect selection transformation strategy with a digital droplet PCR assay designed to detect the presence of the PAT selectable marker, we were also able to identify *T*_1_ plants that carried *AHAS* gene edits and indel mutations but lacked the PAT selectable marker. These results confirmed that selectable marker‐free plants carrying only the desired DNA sequence changes in a nonselected gene can be obtained through genetic segregation.

## Discussion

Several types of engineered nucleases have been developed for the generation of targeted DSBs at precise locations in the genomes of eukaryotes. These DSBs can be exploited for genome engineering using the NHEJ and HDR pathways to create loss‐of‐function alleles via gene knockout, gain or loss of function by gene editing and site‐specific transgene insertion. In this study, we demonstrated the successful application of ZFN technology to generate multiple gene knockouts and gene edits at endogenous loci in bread wheat. We report for the first time in wheat the generation of plants with modified phenotypes via native gene editing using a supplied DNA repair template and transformation strategies based on direct and indirect selection for the targeted trait modification.

The *AHAS*(S653N) mutation conferring resistance to imidazolinone herbicides provides a useful target to investigate different approaches for native gene editing in wheat, because a single copy of this gain‐of‐function allele is sufficient to confer an imazamox‐resistant phenotype. Our results show that gene editing at multiple *AHAS* loci can be recovered in a single plant by transformation methods based on both direct and indirect selection. From transformations based on direct selection for imazamox resistance, we recovered multiple independent wheat plants having mono‐ and biallelic *AHAS* gene editing for the S653N mutation in one or more subgenomes (Table 4). Similarly, from cotransformations based on indirect selection for the *AHAS*(S653N) mutation, where phosphinothricin was used as the selection agent, we were able to recover wheat plants having the desired *AHAS* gene‐editing outcome in one or two subgenomes (Table 5).

Gene duplication and polyploidy in many agriculturally important crops presents a significant challenge for precision genome engineering when all copies of a native gene need to be modified to produce a desired phenotype. For example, ablation of all three homoeoalleles that encode the mildew resistance locus (MLO) proteins in bread wheat was required to obtain plants that had broad‐spectrum resistance to powdery mildew (Wang *et al*., 2014). Traditional mutagenesis methods based on biological, chemical or physical agents such as transfer DNA (T‐DNA), ethyl methane sulphonate and irradiation produce multiple random mutations in the plant genome. These methods require extensive screening of large populations, followed by multiple rounds of backcrossing to identify and isolate the specific mutation causing a desired phenotype. Targeted genome editing using engineered nucleases eliminates the need to purify desired mutations via backcrossing. Our results demonstrate both of these benefits and show the potential for ZFNs to fast‐track forward genetic screens and breeding in wheat. Our results show that cotransformation with a selectable marker and appropriate gene‐editing reagents allows for relatively efficient recovery of gene‐edited wheat plants. From 170 stably transformed events, we recovered two (1.2%) plants with gain‐of‐function alleles and five (2.9%) plants with loss‐of‐function alleles in one or more copies of the targeted native genes (Table 5). Our recovery of wheat plants collectively having targeted modification across all of the *AHAS* genes illustrates the time savings that genome‐editing technologies can provide, even if additional intercrossing is required to introgress edits for multiple genes into a single plant to produce a desired phenotype. Importantly, our results demonstrate it should be possible to modify native genes for any agronomic trait in wheat.

In this study, we focused on utilizing the NHEJ pathway to introduce the S653N mutation into the native *AHAS* genes in wheat via allele insertion and allele replacement. There are several potential advantages for using NHEJ (Hiom, 2010): it is the main pathway for DSB repair in higher eukaryotes; it is active throughout the cell cycle; and it is at least 6‐times faster than HR‐directed repair, which is mainly active in the late S and G2 phase of the cell cycle. Consequently, we assumed NHEJ might be more efficient for gene insertion and gene replacement. Further, NHEJ repair of DSBs with compatible overhangs is reported to be twice as fast as the repair of DSBs with incompatible ends (Mao *et al*., 2008). We therefore hypothesized that ZFN‐mediated NHEJ‐directed gene editing using double‐stranded DNA donor having protruding ends compatible to those at the DSB would produce high‐fidelity repair, resulting in seamless (perfect) donor integration (Figure 4a). High‐fidelity NHEJ repair at both junctions is particularly desirable to avoid frameshift mutations when performing gene insertion or replacement within exons. Deep amplicon sequencing data generated for transient assays performed using these donors suggested the majority of integrated donor had perfect 5′ and 3′ junctions (Table 1). This observation was further supported when we looked at *AHAS* alleles with integrated donor in the *T*_0_ wheat plants recovered from both transformation methods, where we found 63% (15/24) had 3′ junctions with perfect repair. As the 3′ junctions are beyond the *AHAS* stop codon in the donor sequence and are not expected to be under direct selection, we suggest that double‐stranded DNA donor with overhangs compatible to those at the DSB can be used to reduce unwanted indels at the 5′ and 3′ junctions caused by error‐prone NHEJ. Clearly, further work is required to substantiate this statement, for example by comparing donors with and without overhangs compatible to those at the DSB. Our current research is focused on gaining a better understanding for how donor design can be used to fully exploit the NHEJ pathway for genome editing in plants.

Our results show reasonably efficient recovery of wheat plants having *AHAS* allele insertion. Overall, we recovered 13 and two *T*_0_ wheat plants with targeted allele insertion in one or more *AHAS* homoeoalleles from transformations based on direct and indirect selection for imazamox resistance, corresponding to a plant recovery rate of 0.83 and 0.27 per 1000 scutella, respectively. Given that one person can transform more than 1000 scutella per week using particle bombardment, our results indicate it should be possible to produce wheat plants having targeted allele insertion at any trait locus. However, the scale of experiment required to achieve a specific allele insertion outcome will vary depending the position of ZFN cut site within the target gene and length of the DNA repair template. In contrast, we were unsuccessful at regenerating wheat plants with *AHAS* allele replacement. Li *et al*. (2016) reported a 2% (8/390) recovery of *T*_0_ plants with CRISPR/Cas9‐mediated NHEJ‐directed allele replacement at the native EPSPS gene in rice. In that study, they used two CRISPR nucleases cutting the introns flanking EPSPS exon 2 to replace it with an exon carrying mutations conferring glyphosate resistance. Moderate (5%–25%) recovery of *T*_0_ plants having the deletion of intervening sequence between two targeted DSBs has also been reported in other studies in rice (Shan *et al*., 2015; Xie *et al*., 2015; Zhou *et al*., 2014). As the deep amplicon sequencing data from our transient assays indicated allele replacement occurs about sixfold less frequently than allele insertion (average 36% vs 6% of reads showed evidence for donor integration as a percentage of the total number of non‐wild‐type reads; Table 1), it is likely that a larger transformation effort would be required to recover wheat plants with targeted allele replacement. The observed lower frequency for allele replacement in the transient assays, as well as our failure to recover wheat plants with allele replacement, might be expected based on the requirement for a second DSB before replacement of the intervening sequence by the exogenously supplied DNA template could take place. In contrast, allele insertion requires only the creation of a single DSB for integration of the supplied donor to occur.

Both HR and NHEJ have been utilized with engineered nucleases in plants to mediate native gene editing, gene replacement and site‐specific transgene insertion. While it is difficult to directly compare studies to determine whether one pathway might be more efficient than the other (due to differences in the activity of nucleases, and targeting of different genomic loci which themselves may significantly affect frequencies for gene editing and insertion), the rate of recovery of plants with targeted modifications derived from NHEJ‐ or HR‐directed DNA repair using a donor template is low, typically ranging from 0.3% to 15% with a median of 1.5% (D'Halluin *et al*., 2013; Li *et al*., 2016; Shukla *et al*., 2009; Svitashev *et al*., 2015; Wang *et al*., 2014; Zhu *et al*., 2017). On this basis, the recovery rate for plants with targeted gene insertion achieved in our study is within the range reported for major crop species. Specific studies comparing HR‐ and NHEJ‐directed editing outcomes for the same loci and using the same engineered nucleases will be required to fully address whether one pathway is better than the other.

In conclusion, we have demonstrated for the first time in wheat, targeted native gene modification via the NHEJ pathway using ZFNs and a supplied DNA repair template. This strategy was successfully used to introduce the stably inherited S653N mutation into AHAS to confer resistance to the imidazolinone class of herbicides. Further, we demonstrated a general approach for targeted gene modification at nonselectable loci. Our results show the potential for ZFNs to accelerate the study of gene function and the improvement of crop traits. Importantly, this technology is suitable for polyploid species.

## Experimental procedures

### AHAS gene sequencing

PCR primers were designed (Fwd 5′ AYC AGA TGT GGG CGG CTC AGT AT and Rvs 5′ GGG ATA TGT AGG ACA AGA AAC TTG CAT GA) using published genomic sequences from GenBank (Accession Numbers AY210405.1, AY210407.1, AY210406.1, AY210408.1, FJ997628.1, FJ997629.1, FJ997631.1, FJ997630.1, FJ997627.1, AY273827.1) to amplify the homoeologous copies of the *AHAS* genes from *Triticum aestivum* genotype Bobwhite MPB26RH, the donor material used for wheat transformation. The PCR products were purified and cloned using pGEM‐T Easy Vector (Promega) into *E. coli* JM109 cells. Plasmid DNA was extracted using a DNAeasy Plasmid DNA Purification Kit (Qiagen) and Sanger sequenced using BigDye v3.1 chemistry (Applied Biosystems) on an ABI3730XL automated capillary electrophoresis platform. Sequence analysis was performed using Sequencher software (GeneCodes).

### ZFN design and construct assembly

Zinc finger proteins directed against the amplified DNA sequences for the homoeologous copies of the *AHAS* genes were designed as described in Urnov *et al*. (2005). Target binding sites for the designed zinc fingers are shown in Table S1. The *AHAS* targeting zinc finger designs were incorporated into zinc finger expression vectors encoding a protein having at least one finger with a CCHC structure (see US Patent Publication No. 2008/0182332 for further detail). In particular, the last finger in each protein had a CCHC backbone for the recognition helix. The noncanonical zinc finger‐encoding sequences were fused to the nuclease domain of the type IIS restriction enzyme *Fok*I (corresponding to amino acids 384–579 of the sequence of Wah *et al*., 1998) via a four amino acid ZC linker and an opaque‐2 nuclear localization signal derived from *Zea mays* to form zinc finger nucleases. Expression of the ZFN proteins was driven by a constitutively expressed ubiquitin gene (Ubi‐1) promoter, including the 5′ untranslated region, from *Z. mays* (Toki *et al*., 1992) and flanked by the 3′ UTR (comprising the transcriptional terminator and polyadenylation site) from a peroxidase (Per5) gene from *Z. mays* (US Patent Publication No. 2004/0158887). The self‐hydrolysing 2A encoding nucleotide sequence from *Thosea asigna* virus (Szymczak *et al*., 2004) was added between the two ZFN monomer protein coding sequences generating a fusion gene that contains both monomers. Plasmid vectors were assembled using In‐Fusion Advantage Technology (Clontech). Plasmid DNA for each ZFN construct for delivery to wheat cells was prepared from cultures of *E. coli* using the Pure Yield Plasmid Maxiprep System (Promega), following the supplier's instructions.

### Donor synthesis

Each DNA strand of the donor templates DAS000149, DAS000152, DAS000267 and DAS000268 was synthesized as gBlock gene fragments (Integrated DNA Technologies). Double‐stranded donor was prepared by mixing equimolar amounts of each DNA strand, heating at 95 °C for 1 min and cooling to room temperature.

### Protoplast transient assays for assessing ZFN efficacy and specificity

Mesophyll protoplasts from the wheat genotype Bobwhite MPB26RH were prepared for transfection using polyethylene glycol (PEG)‐mediated DNA delivery as follows.

Etiolated leaf material harvested from seed germinated and grown in the dark at 24 °C for 21–28 days was submersed in about 10 mL of leaf digest mix (0.6 m mannitol, 10 mm MES, 1.5% w/v cellulase R10, 0.3% w/v macerozyme, 1 mm CaCl_2_, 1% bovine serum albumin, 0.025% v/v pluronic acid, 5 mm β‐mercaptoethanol, pH 5.7) and sliced transversely into 1‐ to 2‐mm segments using a scalpel blade. Additional leaf digest mix was added to a final volume of 10 mL per gram fresh weight of leaf material and subjected to vacuum (20″ Hg) pressure for 30 min, followed by incubation at 28 °C with rotational shaking at 30 r.p.m. for 4 h. Mesophyll protoplasts released from the leaf material were collected by passing the digestion suspension through a 100‐μm sieve, followed by 70‐ and 40‐μm sieves. The digested leaf material was washed three times with 10 mL wash buffer (20 mm KCl, 4 mm MES, 0.6 m mannitol, pH 5.6) to maximize protoplast yield. The filtered protoplasts were pelleted by centrifugation at 70 ***g*** and 15 °C for 10 min and the supernatant removed. The protoplast pellet was washed once in 7 mL of wash buffer and finally resuspended in 1 mL wash buffer. The yield of mesophyll protoplasts was estimated using a Neubauer haemocytometer. Evans Blue stain was used to determine the proportion of live cells.

PEG‐mediated DNA transfection was performed by pelleting about 1 million protoplasts by centrifugation at 70 ***g*** and 15 °C for 10 min. The protoplasts were resuspended in 600 μL of wash buffer containing 70 μg of plasmid DNA, comprising 30 μg of a ZFN construct and 40 μg of a reporter construct for expression of red fluorescent protein (DsRed). An equal volume of 40% PEG solution (40% w/v PEG 4000, 0.2 m mannitol, 0.1 m Ca(NO_3_)_2_, pH 5.6) was slowly added to the protoplast suspension with simultaneous mixing by gentle rotation of the tube. The protoplast suspension was allowed to incubate for 15 min at room temperature occasional gentle mixing. Six millilitres of wash buffer was then slowly added to the protoplast suspension, with simultaneous gentle mixing to maintain a homogenous suspension. The protoplasts were pelleted by centrifugation at 70 ***g*** and 15 °C for 10 min and the supernatant removed. The protoplast pellet was resuspended in 2 mL wash buffer before an additional 7 mL of wash buffer was added. Centrifugation was repeated to pellet to the protoplasts and the supernatant removed. The protoplast pellet was resuspended in 2 mL Qiao's media (0.44% w/v MS plus vitamins, 3 mm MES, 0.0001% w/v 2,4‐D, 0.6 m glucose, pH 5.7) and transferred to a sterile 3‐cm Petri dish before incubation in the dark for 24 °C for 72 h. The transfection efficiency was determined using FACS (BD‐Biosciences), as the proportion of protoplasts having red fluorescent protein expression.

Genomic DNA was extracted from the transfected mesophyll protoplasts using the DNAeasy Plant DNA Extraction Mini Kit (Qiagen) following the manufacturer's instructions, except that tissue disruption was not required and the protoplasts were added directly to the lysis buffer. The DNA region surrounding the ZFN target site was amplified using two rounds of PCR to enable incorporation of amplicon‐specific barcodes for Illumina sequencing. The primers for the first PCR round were designed to amplify the *AHAS* homoeoloci and to include nucleotide variants that distinguished between *AHAS* gene copies originating from the different wheat subgenomes. These primers were tagged at their 5′ end with sequences to enable Illumina barcode indexing in the subsequent PCR step (AHAS.F1 5′ aca ctc ttt ccc tac acg acg ctc ttc cga tct GAG ACC CCA GGG CCA TAC TTG; AHAS.R3 5′ gtg act gga gtt cag acg tgt gct ctt ccg atc tCA AGC AAA CTA GAA AAC GCA TGG, where lower case indicates Illumina sequences). The second round of PCR was performed using Illumina's commercially available indexing primers. The resulting amplicons were purified using Agencourt Ampure XP magnetic beads (Beckman‐Coulter) with a DNA‐to‐bead ration of 1:1.7. The purified amplicons were titrated for sequencing by Illumina short read technology using a PCR‐based library quantification kit (KAPA) according the manufacturer's instructions (KAPA Biosystems). The samples were sequenced on an Illumina GAII_x_ or HiSeq2000 instrument (Illumina) according to the manufacturer's instructions.

Zinc finger nuclease efficacy was determined as the proportion of sequence reads arising from each *AHAS* homoeolocus that contained NHEJ‐derived indels. The observed efficacy was expressed in parts per thousand (ppk) activity after normalizing for protoplast transfection efficiency. Only reads with >1‐nucleotide indel within a 10‐bp window centred over the expected site of cleavage were classified as NHEJ mutations. The frequency distribution of indel sizes was determined from the most prevalent NHEJ mutations arising within the expected cleavage site, and the predominant overhang generated by each ZFN determined from the most common mutation. The ranking order of the ZFNs by their observed cleavage efficiency was used to select ZFNs with the best cleavage activity for subsequent experiments.

### Scutella transient assays for assessing NHEJ‐directed gene‐editing efficiencies

Twenty scutella of immature zygotic wheat embryos from the donor genotype Bobwhite MPB26RH were cobombarded with each ZFN vector and donor template combination. Briefly, for allele insertion, DNA for donor DAS000267 (or DAS000152) was mixed at a 5:1 molar ratio with plasmid DNA for ZFN DAB109350, and for allele replacement, DNA for donor DAS000268 (or DAS000149) was mixed at a 5:1:1 molar ratio with plasmid DNA for ZFNs DAB109350 and DAB109360. DNA was precipitated onto gold particles for biolistic‐mediated DNA delivery as follows: 50 μL of 40 mg/mL of 0.6 μm colloidal gold particles (Bio‐Rad) was added to 5 μL of DNA solution (1 μg DNA/μL), followed by sequential addition of 50 μL of 2.5 M CaCl_2_ and 20 μL of 0.1 m spermidine. The mixture was vortexed for 1 min. The DNA‐coated gold particles were pelleted by centrifugation and the supernatant removed. The gold particles were washed once in 1 mL of absolute ethanol and resuspended in 100 μL of absolute ethanol. Immediately following resuspension, 10 μL of DNA‐coated gold particles was placed centrally onto a macrocarrier membrane and allowed to air dry. Particle bombardment was performed using the PDS‐100/HE Particle Gun Delivery System (Bio‐Rad) with a 900 psi rupture disc and the following settings: gap 2.5 cm, stopping plate aperture 0.8 cm, target distance 6.0 cm, vacuum 91.4–94.8 kPa, vacuum flow rate 5.0 and vent flow rate 4.5. Postbombardment the scutella were cultured on callus induction media in the dark at 26 °C for 7 days. Genomic DNA was isolated from the 20 scutella using the DNAeasy Plant DNA Extraction Mini Kit (Qiagen) according to the manufacturer's instructions. The DNA region surrounding the ZFN target site was amplified using two rounds of PCR and subjected to deep amplicon sequencing, as described above. The frequency of ZFN‐induced gene editing was assessed as the proportion of sequence reads arising from each *AHAS* homoeolocus having donor integration. The proportion of sequence reads having NHEJ‐derived mutation was calculated as described above.

### Wheat transformation

Particle bombardment transformation of scutella from immature zygotic embryos from the donor genotype Bobwhite MPB26RH was used to generate gene‐edited wheat plants as described above. Postbombardment the scutella were cultured in the dark on callus induction medium at 24 °C for 2 weeks. The resultant calli were subcultured once onto fresh callus induction medium and kept in the same conditions for a further 2 weeks. Somatic embryogenic callus (SEC) was transferred onto plant regeneration medium and cultured for 2 weeks at 24 °C under a 16/8‐h (light/dark) hour photoperiod in a growth room. Regenerated plantlets were transferred onto rooting medium and cultured under the same conditions for 2–3 weeks. Chemical selection agent (imazamox or phosphinothricin) was added to each culture medium as described in the *Results* section. To increase stringency for the selection of regenerated plants having the S653N mutation, the roots of regenerated plants were removed and the plants were again subcultured on rooting media containing imazamox under the same conditions. Plantlets rooting after a second round of rooting media were transferred to compost‐based potting mix and grown under glasshouse containment conditions. *T*_1_ seed was harvested from individual plants, following bagging of individual spikes prior to flowering to prevent out‐crossing.

### Characterization of AHAS‐edited wheat plants and Mendelian inheritance

PCR was used to characterize the subgenomic location, zygosity and outcomes for ZFN‐mediated gene editing in the transformed wheat plants. The DNA region surrounding the ZFN target sites were amplified using the AHAS.F1/AHAS.R3 primer pair (see above) but without the 5′ Illumina barcode indexing sequences. The resulting amplicons were separated and visualized on 2% agarose gel, or cloned into pGEM‐T plasmid vector (Promega) and Sanger sequenced as described above. Up to 120 independent plasmid clones were sequenced to ensure each *AHAS* homoeoallele was characterized. Sequencher^®^ software (GeneCodes) was used to generate a consensus sequence for each *AHAS* homoeoallele and to determine its subgenomic origin.

The Bio‐Rad QX100™ Droplet Digital PCR (ddPCR) system (Bio‐Rad) was used to quantitatively assess the number of wild‐type, NHEJ indel and donor‐integrated *AHAS* alleles present in the transformed wheat plants and their offspring. ddPCR increases the sensitivity and dynamic range of quantitative PCR by separating each reaction into a large number of noninteracting partitions (Mazaika and Homsy, 2014). A TaqMan™ assay was performed using two ZEN™ Double Quenched hydrolysis probes (Integrated DNA Technologies) that were positioned within the same amplicon. The first hydrolysis probe (5′ FAM‐CCA GGG CCA TAC TTG TTG GAT ATC) detected the presence of any *AHAS* homoeoallele, while the second probe (5′ HEX‐TTG GAA TCA TAG GCA GCA CGT) was positioned over the ZFN cut site and designed to discriminate between wild‐type, NHEJ mutations and donor integration. The forward and reverse primer pair for this dual TaqMan assay were 5′ GTG ACG AAG AAG AGC GAA G and 5′ GCC ATC ACC CTC CATG AT, respectively. PCR droplets were classified as wild‐type, NHEJ‐derived indel or S653N edited based on the ratio of HEX:FAM using QuantaSoft software (Bio‐Rad). To ensure correct droplet classification, control assays were performed using synthetic DNA templates corresponding to the expected wild‐type, NHEJ indel and S653N donor‐integrated alleles. A duplex TaqMan digital droplet PCR assay was used to detect the presence of the PAT selectable marker. This assay comprised a primer pair (Fwd 5′ GAT CAA TTG TTC GAC AGT GAA GGT, Rvs 5′ TCA AAA TGA CTG GCC TAA TCA GAT AA) and ZEN™ Double Quenched hydrolysis probe (5′ HEX‐TCC GCT GGG CAT AAT TCC AAT GAG C) to amplify the known endogenous single copy gene, EST BE497897, from chromosome 6B of hexaploid wheat (Qi *et al*., 2004) and a primer pair (Fwd 5′ CCC GCC CCT CTC CTC TTT C, Rvs 5′ CTC CCG CGC ACC GAT CTG) and ZEN™ Double Quenched hydrolysis probe (5′ FAM‐AAG CCG CCT CTC GCC CAC CCA) targeting a region of the *Oryza sativa* Actin (Act1) promoter present in a wheat codon optimized version of the phosphinothricin acetyl transferase gene (PAT). The number of copies of the exogenous PAT gene was determined using the CNV functionality in the QuantaSoft software.

## Conflict of interest

The authors declare that they have no conflict of interest.

## Supporting information


**Table S1** Target sites for AHAS zinc fingers.
**Table S2** Chi‐square analyses for expected Mendelian inheritance in the *T*_1_ generation.
