# Supplementary material for: Zinc finger nuclease‐mediated precision genome editing of an endogenous gene in hexaploid bread wheat (Triticum aestivum) using a DNA repair template
Source: Plant Biotechnol J. 2018 May 28;16(12):2088–101. doi: 10.1111/pbi.12941 (PMC6230953; doi:10.1111/pbi.12941)
Supplement: Supplementary file 1 — Table S1 Target sites for AHAS zinc fingers. Table S2 Chi‐square analyses for expected Mendelian inheritance in the T 1 generation. [file PBI-16-2088-s001.docx]

**Supplementary Table 1**. Target sites for AHAS zinc fingers. Nucleotides in the target site that are contacted by the zinc finger protein recognition helices are indicated in uppercase letters; non-contacted nucleotides are indicated in lowercase.

| **Vector** | **ZFP1** | **Target Site (5’->3’)** | **ZFP2** | **Target Site (5’->3’)** |
| --- | --- | --- | --- | --- |
| DAB109350 | 29732 | gaTCCCAAGCGGTGGTGctttcaaggac | 29730 | agGCAGCACGTGCTCCTGATgcgggact |
| DAB109351 | 29732 | gaTCCCAAGCGGTGGTGctttcaaggac | 29731 | taGGCAGCACGtgCTCCTGatgcgggac |
| DAB109352 | 29753 | tcTTGTAGGTCGAAatttcagtacgagg | 29754 | ctACAAGTGTGaCATGCGcaatcagcat |
| DAB109359 | 30006 | tgATGCGGGACTATGATatccaacaagt | 30008 | gaGCACGTGCTgCCTATGatcccaagcg |
| DAB109360 | 30012 | tcTTGTAGGTCGAAATTtcagtacgagg | 30018 | taCAAgTGTGACaTGCGCAatcagcatg |
| DAB109361 | 30014 | tcTTGTAGGTCGAAATTtcagtacgagg | 30018 | taCAAgTGTGACaTGCGCAatcagcatg |
| DAB109385 | 29770 | cAAGTGTGACaTGCGCAa | 29769 | cTTGTAGGTCGAAa |

**Supplementary Table 2**. Chi-square analyses for expected Mendelian inheritance in the T_1_ generation. Twenty four T_1_ plants derived from selfing each T_0_ event were assessed using a digital droplet PCR assay designed to measure the number of wild-type, indel and donor-integrated AHAS alleles present in each plant.

| Event |  | T0 genotype | No. T1 expected | | | No. T1 observed | | | χ2 (1:2:1) |
| --- | --- | --- | --- | --- | --- | --- | --- | --- | --- |
| 1 | A-genome | AO/KO | AO/AO | AO/KO | KO/KO | AO/AO | AO/KO | KO/KO |  |
|  |  |  | 6 | 12 | 6 | 6 | 15 | 3 | 2.25 |
|  | B-genome | AI/WT | AI/AI | AI/WT | WT/WT | AI/AI | AI/WT | WT/WT |  |
|  |  |  | 6 | 12 | 6 | 3 | 13 | 8 | 2.25 |
|  | D-genome | AI/WT | AI/AI | AI/WT | WT/WT | AI/AI | AI/WT | WT/WT |  |
|  |  |  | 6 | 12 | 6 | 9 | 13 | 2 | 4.25 |
| 2 | A-genome | KO/WT | KO/KO | KO/WT | WT/WT | KO/KO | KO/WT | WT/WT |  |
|  |  |  | 6 | 12 | 6 | 3 | 15 | 6 | 2.25 |
|  | B-genome | WT/WT | WT/WT | . | . | WT/WT | . | . |  |
|  |  |  | 24 | . | . | 24 | . | . | na |
|  | D-genome | AI/WT | AI/AI | AI/WT | WT/WT | AI/AI | AI/WT | WT/WT |  |
|  |  |  | 6 | 12 | 6 | 9 | 10 | 5 | 2.00 |
| 3 | A-genome | AI/WT | AI/AI | AI/WT | WT/WT | AI/AI | AI/WT | WT/WT |  |
|  |  |  | 6 | 12 | 6 | 8 | 9 | 7 | 1.58 |
|  | B-genome | WT/WT | WT/WT | . | . | WT/WT | . | . |  |
|  |  |  | 24 | . | . | 24 | . | . | na |
|  | D-genome | WT/WT | WT/WT | . | . | WT/WT | . | . |  |
|  |  |  | 24 | . | . | 24 | . | . | na |
| 4 | A-genome | AI/WT | AI/AI | AI/WT | WT/WT | AI/AI | AI/WT | WT/WT |  |
|  |  |  | 6 | 12 | 6 | 6 | 14 | 4 | 1.00 |
|  | B-genome | AI/WT | AI/AI | AI/WT | WT/WT | AI/AI | AI/WT | WT/WT |  |
|  |  |  | 6 | 12 | 6 | 9 | 8 | 7 | 3.00 |
|  | D-genome | AI/AI | AI/AI | . | . | AI/AI | . | . |  |
|  |  |  | 24 | . | . | 24 | . | . | na |
| 5 | A-genome | AI/WT | AI/AI | AI/WT | WT/WT | AI/AI | AI/WT | WT/WT |  |
|  |  |  | 6 | 12 | 6 | 10 | 7 | 7 | 4.92 |
|  | B-genome | KO/WT | KO/KO | KO/WT | WT/WT | KO/KO | KO/WT | WT/WT |  |
|  |  |  | 6 | 12 | 6 | 6 | 9 | 9 | 2.25 |
|  | D-genome | WT/WT | WT/WT | . | . | WT/WT | . | . |  |
|  |  |  | 24 | . | . | 24 | . | . | na |
| 6 | A-genome | WT/WT | WT/WT | . | . | WT/WT | . | . |  |
|  |  |  | 24 | . | . | 24 | . | . | na |
|  | B-genome | AI/WT | AI/AI | AI/WT | WT/WT | AI/AI | AI/WT | WT/WT |  |
|  |  |  | 6 | 12 | 6 | 4 | 15 | 5 | 1.58 |
|  | D-genome | WT/WT | WT/WT | . | . | WT/WT | . | . |  |
|  |  |  | 24 | . | . | 24 | . | . | na |
| 7 | A-genome | AI/WT | AI/AI | AI/WT | WT/WT | AI/AI | AI/WT | WT/WT |  |
|  |  |  | 6 | 12 | 6 | 2 | 12 | 10 | 5.33 |
|  | B-genome | WT/WT | WT/WT | . | . | WT/WT | . | . |  |
|  |  |  | 24 | . | . | 24 | . | . | na |
|  | D-genome | WT/WT | WT/WT | . | . | WT/WT | . | . |  |
|  |  |  | 24 | . | . | 24 | . | . | na |
| 8 | A-genome | WT/WT | WT/WT | . | . | WT/WT | . | . |  |
|  |  |  | 24 | . | . | 24 | . | . | na |
|  | B-genome | WT/WT | WT/WT | . | . | WT/WT | . | . |  |
|  |  |  | 24 | . | . | 24 | . | . | na |
|  | D-genome | WT/WT | WT/WT | . | . | WT/WT | . | . |  |
|  |  |  | 24 | . | . | 24 | . | . | na |
| 9 | A-genome | AI/AI | AI/AI | . | . | AI/AI | . | . |  |
|  |  |  | 24 | . | . | 24 | . | . | na |
|  | B-genome | AI/WT | AI/AI | AI/WT | WT/WT | AI/AI | AI/WT | WT/WT |  |
|  |  |  | 6 | 12 | 6 | 5 | 17 | 2 | 4.92 |
|  | D-genome | WT/WT | WT/WT | . | . | WT/WT | . | . |  |
|  |  |  | 24 | . | . | 24 | . | . | na |
| 10 | A-genome | AI/WT | AI/AI | AI/WT | WT/WT | AI/AI | AI/WT | WT/WT |  |
|  |  |  | 6 | 12 | 6 | 2 | 16 | 6 | 4.00 |
|  | B-genome | WT/WT | WT/WT | . | . | WT/WT | . | . |  |
|  |  |  | 24 | . | . | 24 | . | . | na |
|  | D-genome | WT/WT | WT/WT | . | . | WT/WT | . | . |  |
|  |  |  | 24 | . | . | 24 | . | . | na |
| 11 | A-genome | WT/WT | WT/WT | . | . | WT/WT | . | . |  |
|  |  |  | 24 | . | . | 24 | . | . | na |
|  | B-genome | WT/WT | WT/WT | . | . | WT/WT | . | . |  |
|  |  |  | 24 | . | . | 24 | . | . | na |
|  | D-genome | AI/WT | AI/AI | AI/WT | WT/WT | AI/AI | AI/WT | WT/WT |  |
|  |  |  | 6 | 12 | 6 | 7 | 11 | 6 | 0.25 |
| 12 | A-genome | WT/WT | WT/WT | . | . | WT/WT | . | . |  |
|  |  |  | 24 | . | . | 24 | . | . | na |
|  | B-genome | WT/WT | WT/WT | . | . | WT/WT | . | . |  |
|  |  |  | 24 | . | . | 24 | . | . | na |
|  | D-genome | AI/KO | AI/AI | AI/KO | KO/KO | AI/AI | AI/KO | KO/KO |  |
|  |  |  | 6 | 12 | 6 | 7 | 15 | 1 | 5.08 |
| 13 | A-genome | AI/WT | AI/AI | AI/WT | WT/WT | AI/AI | AI/WT | WT/WT |  |
|  |  |  | 6 | 12 | 6 | 6 | 14 | 4 | 1.00 |
|  | B-genome | WT/WT | WT/WT | . | . | WT/WT | . | . |  |
|  |  |  | 24 | . | . | 24 | . | . | na |
|  | D-genome | AI/WT | AI/AI | AI/WT | WT/WT | AI/AI | AI/WT | WT/WT |  |
|  |  |  | 6 | 12 | 6 | 7 | 11 | 6 | 0.25 |
| 14 | A-genome | AI/WT | AI/AI | AI/WT | WT/WT | AI/AI | AI/WT | WT/WT |  |
|  |  |  | 6 | 12 | 6 | 3 | 15 | 6 | 2.25 |
|  | B-genome | WT/WT | WT/WT | . | . | WT/WT | . | . |  |
|  |  |  | 24 | . | . | 24 | . | . | na |
|  | D-genome | WT/WT | WT/WT | . | . | WT/WT | . | . |  |
|  |  |  | 24 | . | . | 24 | . | . | na |
| 15 | A-genome | WT/WT | WT/WT | . | . | WT/WT | . | . |  |
|  |  |  | 24 | . | . | 24 | . | . | na |
|  | B-genome | AI/WT | AI/AI | AI/WT | WT/WT | AI/AI | AI/WT | WT/WT |  |
|  |  |  | 6 | 12 | 6 | 5 | 17 | 2 | 4.92 |
|  | D-genome | WT/WT | WT/WT | . | . | WT/WT | . | . |  |
|  |  |  | 24 | . | . | 24 | . | . | na |
| 16 | A-genome | AI/AI | AI/AI | . | . | AI/AI | . | . |  |
|  |  |  | 24 | . | . | 24 | . | . | na |
|  | B-genome | WT/WT | WT/WT | . | . | WT/WT | . | . |  |
|  |  |  | 24 | . | . | 24 | . | . | na |
|  | D-genome | WT/WT | WT/WT | . | . | WT/WT | . | . |  |
|  |  |  | 24 | . | . | 24 | . | . | na |
| 17 | A-genome | KO/WT | KO/KO | KO/WT | WT/WT | KO/KO | KO/WT | WT/WT |  |
|  |  |  | 6 | 12 | 6 | 7 | 11 | 6 | 0.25 |
|  | B-genome | WT/WT | WT/WT | . | . | WT/WT | . | . |  |
|  |  |  | 24 | . | . | 24 | . | . | na |
|  | D-genome | WT/WT | WT/WT | . | . | WT/WT | . | . |  |
|  |  |  | 24 | . | . | 24 | . | . | na |
| 18 | A-genome | KO/WT | KO/KO | KO/WT | WT/WT | KO/KO | KO/WT | WT/WT |  |
|  |  |  | 6 | 12 | 6 | 5 | 17 | 2 | 4.92 |
|  | B-genome | WT/WT | WT/WT | . | . | WT/WT | . | . |  |
|  |  |  | 24 | . | . | 24 | . | . | na |
|  | D-genome | WT/WT | WT/WT | . | . | WT/WT | . | . |  |
|  |  |  | 24 | . | . | 24 | . | . | na |
| 19 | A-genome | KO/WT | KO/KO | KO/WT | WT/WT | KO/KO | KO/WT | WT/WT |  |
|  |  |  | 6 | 12 | 6 | 6 | 11 | 7 | 0.25 |
|  | B-genome | WT/WT | WT/WT | . | . | WT/WT | . | . |  |
|  |  |  | 24 | . | . | 24 | . | . | na |
|  | D-genome | WT/WT | WT/WT | . | . | WT/WT | . | . |  |
|  |  |  | 24 | . | . | 24 | . | . | na |
| 20 | A-genome | KO/WT | KO/KO | KO/WT | WT/WT | KO/KO | KO/WT | WT/WT |  |
|  |  |  | 6 | 12 | 6 | 8 | 13 | 3 | 2.25 |
|  | B-genome | WT/WT | WT/WT | . | . | WT/WT | . | . |  |
|  |  |  | 24 | . | . | 24 | . | . | na |
|  | D-genome | WT/WT | WT/WT | . | . | WT/WT | . | . |  |
|  |  |  | 24 | . | . | 24 | . | . | na |
| 21 | A-genome | WT/WT | WT/WT | . | . | WT/WT | . | . |  |
|  |  |  | 24 | . | . | 24 | . | . | na |
|  | B-genome | KO/WT | KO/KO | KO/WT | WT/WT | KO/KO | KO/WT | WT/WT |  |
|  |  |  | 6 | 12 | 6 | 9 | 11 | 4 | 2.25 |
|  | D-genome | KO/WT | KO/KO | KO/WT | WT/WT | KO/KO | KO/WT | WT/WT |  |
|  |  |  | 6 | 12 | 6 | 3 | 12 | 9 | 3.00 |
